# BMI Modulates the Effect of Thyroid Hormone on Lipid Profile in Euthyroid Adults

**DOI:** 10.1155/2017/8591986

**Published:** 2017-08-13

**Authors:** Yanqiu Wang, Qinglei Yin, Min Xu, Qicheng Ni, Weiqing Wang, Qidi Wang

**Affiliations:** ^1^Shanghai National Clinical Research Center for Endocrine and Metabolic Diseases, Key Laboratory for Endocrine and Metabolic Diseases of Chinese Health Ministry, Ruijin Hospital Affiliated to Shanghai Jiaotong University School of Medicine, Shanghai 200025, China; ^2^Sino-French Research Center for Life Sciences and Genomics, Ruijin Hospital Affiliated to Shanghai Jiaotong University School of Medicine, Shanghai 200025, China

## Abstract

The impacts of thyroid hormones (TH) on lipid profile in euthyroid adults have gained much attention. It is currently unknown whether BMI influences such interaction. In the present study, we investigate the role of BMI in modulating the association between TH and lipid parameters in 1372 euthyroid healthy adults. Our results show that thyroid parameters are differentially associated with lipid profile. FT3 is positively correlated with total cholesterol (*β* = 0.176 ± 0.046, *P* < 0.001) and LDL cholesterol levels (*β* = 0.161 ± 0.040, *P* < 0.001). FT4 is negatively correlated with TG (*β* = −0.087 ± 0.029, *P* < 0.01) while positively correlated with HDL cholesterol levels (*β* = 0.013 ± 0.005, *P* < 0.01). TSH is positively associated with TG (*β* = 0.145 ± 0.056, *P* < 0.05) and total cholesterol levels (*β* = 0.094 ± 0.030, *P* < 0.01). Importantly, BMI modulates the effect of TH on lipid profile: the interaction of FT4 and BMI and the interaction of FT3 and BMI reach statistical significance in predicting TG and HDL cholesterol levels, respectively. Stratified according to BMI levels, most associations between TH and lipid profile are significant only in normal-weight group. In conclusion, in euthyroid adults, high normal FT3, TSH levels, and low normal FT4 levels are associated with unfavorable lipid profile. BMI mediates the effect of thyroid function on lipid profile in euthyroid adults.

## 1. Introduction

Dyslipidemia constitutes a major risk factor for premature atherosclerosis and cardiovascular disease (CVD), which is a major cause of morbidity and mortality in both developed and developing countries [1]. Dyslipidemia is defined as increased triglycerides (TG) or low-density lipoprotein cholesterol (LDL cholesterol) levels, or decreased high-density lipoprotein cholesterol (HDL cholesterol) levels. A clustering of risk factors for developing dyslipidemia is age, eating habits, physical activity, stress, and heredity, as well as thyroid function [2].

Thyroid hormones (TH) regulate multiple metabolic processes [3]. It is widely accepted that overt thyroid dysfunctions (both hypo- and hyperthyroidism) are correlated with alternations in lipid and glucose metabolism [2]. Hyperthyroidism leads to a hypermetabolic state and increased lipolysis, which is characterized by weight loss, lower plasma levels of HDL cholesterol, LDL cholesterol, and leptin [2]. Conversely, hypothyroidism has the opposite effects: hypothyroid patients are presented with higher plasma TG, total cholesterol, and LDL cholesterol levels [2, 4, 5]. The mechanisms of thyroid hormone on metabolic phenotype in patients with thyroid disease have been extensively investigated [3, 5]. Recently, the impacts of thyroid hormones within normal ranges on lipid profile have also been clarified [6–10]. In euthyroid adults, FT3 within normal range is positively correlated to dyslipidemia: high normal FT3 is associated with a less favorable metabolic phenotype in pregnant women [10] and in general euthyroid population [6]. On the other hand, several studies have shown that low normal FT4 or high normal TSH levels are associated with unfavorable lipid profile and higher CVD risk [7, 9, 11–14]. Roos et al. found a negative relation between FT4 and plasma total cholesterol and LDL cholesterol levels [7]. Moreover, a negative association between FT4 and TG was also reported in euthyroid adults, even after adjustment for potential confounders [15, 16]. TSH was positively associated with total cholesterol and LDL cholesterol levels both in euthyroid men and women [11, 13]. Interestingly, in obese euthyroid population, Temizkan et al. could not identify significant associations between TSH and atherogenic dyslipidemia [8]. Moreover, in the obese setting, Marzullo et al. reported that TSH was positively associated with total cholesterol levels; however, the association disappeared after controlling for individuals' confounders, including BMI [16]. It is known that thyroid function status, even within normal ranges, might correlate with alternations in body weight and energy metabolism [17–19]. Moreover, it is well documented that obesity is correlated not only with unfavorable metabolic profiles but also with changes in thyroid hormone levels [20]. So far, little is known whether BMI mediates the influence of thyroid hormones on lipid profile in euthyroid subjects.

The objective of the current study is to examine the association between thyroid hormones and lipid parameters in euthyroid adults. Furthermore, the role of BMI in modulating the association between thyroid hormones and lipid profile is determined.

## 2. Methods

### 2.1. Study Population

We conducted this retrospective study with subjects who participated in a routine health screening examination in Ruijin Hospital affiliated to Shanghai Jiao Tong University School of Medicine between 2007 and 2010. Individuals with previously diagnosed chronic cardiovascular diseases, diabetes mellitus, and liver and kidney diseases were excluded from our study. Women with diagnosed pregnancy were also excluded. Furthermore, individuals receiving thyroid hormone substitutions or antithyroid drugs, which may potentially interfere with thyroid function or having TPO antibodies, antithyroglobulin antibodies above the clinical cutoff for positivity, or with TSH levels outside the normal reference range of our laboratory (TSH: 0.35–4.94 mIU/L), were also excluded from our analyses, finally leaving 1372 subjects for inclusion in this investigation.

The study was approved by the Medical Ethics Committee of Ruijin Hospital, Shanghai Jiao Tong University School of Medicine and was conducted in accord with institutional guidelines. The study was in accordance with the principle of the Helsinki Declaration II. All study participants provided written informed consents.

### 2.2. Measurements

Data on gender and age were obtained from all the subjects. Standing height (cm) was measured using a wall-mounted Harpenden Stadiometer. Body weight (kg) was measured in light indoor clothing without shoes. Body mass index is defined as the body weight (kg) divided by the square of the body height (m).

Normal weight is defined as 18.5 ≤ BMI< 25 kg/m^2^, while overweight and obese are defined as BMI ≥ 25 kg/m^2^ according to the WHO criteria [21].

### 2.3. Biochemical Determinations

Blood samples were obtained from all the subjects after overnight fasting. Chemiluminescent methods (Cobas E601; Roche, Basel, Switzerland) were used to quantitate thyroid function based on free triiodothyronine (FT_3_), free thyroxine (FT_4_), and thyrotropin (TSH) levels. Serum total cholesterol, low-density lipoprotein cholesterol (LDL cholesterol), high-density lipoprotein cholesterol (HDL cholesterol), and TG were measured using an autoanalyzer (ARCHITECT ci16200 analyzer; Abbott Laboratories, Abbott, IL) in the Shanghai Institute of Endocrine and Metabolic Diseases.

### 2.4. Statistical Analysis

All statistical analyses (linear regression analyses, logistic regression analyses, and one-way analysis of variance (ANOVA)) were performed using SAS 9.2 (SAS Institute, Cary, NC). Data were given as mean ± standard deviation for normally distributed continuous parameters, and for skewness distribution data, median and interquartile range was used. Descriptive statistics were used to describe the study population at the baseline.

Linear regression analysis was used to identify the association between different thyroid parameters and different lipid parameters. These models were all adjusted for age, gender, and BMI. Each BMI subgroup was sequentially assigned to tertiles according to their plasma FT3, FT4, and TSH levels. Associations between thyroid function parameters and lipid profile among tertiles were identified using one-way ANOVA in each BMI subgroup. *P* for interaction resulted from linear regression analysis with FT3^∗^BMI (FT4^∗^BMI, or TSH^∗^BMI) as independent variable and lipid profile as dependent variables, after multiple adjustment for age, gender, BMI, and FT3 (or FT4 and TSH). We further divided the population into subgroups according to gender, and *P* for interaction was calculated after adjustment for age, BMI, and FT3 (or FT4 and TSH).

The *P* values reported were two sided. Statistical significance was assumed for *P* values < 0.05.

## 3. Results

### 3.1. General Characteristics of the Study Population

Characteristics of the study population, their anthropometric data, metabolic parameters, and descriptions for thyroid hormones were shown in Table 1. A total of 1372 euthyroid subjects (973 males and 399 females) were enrolled in this study, in which 36.0% were overweight (25 ≤ BMI< 30 kg/m^2^) and 4.2% were obese (BMI ≥ 30 kg/m^2^). There were evident differences in lipid parameters and thyroid hormone levels between subgroups with BMI ≥ 25 kg/m^2^ (overweight/obese group: average BMI 27.28 ± 1.96 kg/m^2^) and with 18.5 ≤ BMI< 25 kg/m^2^ (normal-weight group: average BMI 22.43 ± 1.70 kg/m^2^). Subjects in the overweight/obese group had worse lipid profile compared to the normal-weight controls: they had significantly higher TG levels (1.90 (1.32–2.86) versus 1.27 (0.87–1.89) mmol/L, *P* < 0.001), total cholesterol levels (4.95 ± 0.94 versus 4.82 ± 0.96 mmol/L, *P* < 0.05), and lower HDL cholesterol levels (11.64 ± 2.95 versus 13.21 ± 3.34 mg/L, *P* < 0.001) **(**Table 1**)**. There was no significant difference in LDL cholesterol levels between the two groups **(**Table 1**)**.

Thyroid hormone levels between subjects in different BMI categories also varied significantly. Subjects in the overweight/obese group showed increased FT3 levels (4.29 ± 0.58 versus 4.11 ± 0.57 pmol/L, *P* < 0.001), but decreased FT4 levels (12.41 ± 1.54 versus 12.62 ± 1.70 pmol/L, *P* < 0.05). Moreover, TSH levels were slightly lower in the overweight/obese group but did not reach statistical significance (1.58 ± 0.85 versus 1.66 ± 0.86 mIU/L, *P* = 0.074).

### 3.2. The Association between TH and Lipid Profile in Euthyroid Adults

To understand the relationship between the levels of thyroid hormones and lipid parameters, we performed linear regression analyses, adjusted for age, gender, and BMI in euthyroid subjects. As shown in Table 2, FT3 and FT4 were differentially associated with lipid parameters in euthyroid subjects. FT3 was significantly and positively correlated with total cholesterol (*β* = 0.176 ± 0.046, *P* < 0.001) and LDL cholesterol concentrations (*β* = 0.161 ± 0.040, *P* < 0.0001). On the contrary, FT4 levels were negatively correlated with TG (*β* = −0.087 ± 0.029, *P* < 0.01) but positively associated with HDL cholesterol levels (*β* = 0.013 ± 0.005, *P* < 0.01). TSH levels were significantly and positively related to both TG (*β* = 0.145 ± 0.056, *P* < 0.05) and total cholesterol levels (*β* = 0.094 ± 0.030, *P* < 0.01).

### 3.3. BMI Modulates the Association between TH and Lipid Profile in Euthyroid Adults

We further tested whether there were interactions between BMI and thyroid parameters in modulating lipid profile. Importantly, the interaction of FT3 and BMI and the interaction of FT4 and BMI reached statistical significance in predicting serum HDL cholesterol (*P* < 0.05) and TG concentrations (*P* < 0.05), respectively, after multiple adjustment for age, gender, BMI, and FT3 (or FT4) (Table 3). We also examined the interaction of TSH and BMI in predicting lipid parameters; however, we could not find statistical significance in predicting any of the parameters in the whole population (Table 3).

It is known that thyroid function and lipid profile can be influenced by gender [6, 12, 14]. Therefore, gender-based interaction analyses were also performed. As shown in Table S1 available online at https://doi.org/10.1155/2017/8591986, the interaction of FT3 and BMI remained significant in predicting serum HDL cholesterol in male subjects (*P* < 0.05), whereas the interaction of FT4 and BMI was significant in modulating TG concentrations in females (*P* < 0.05). Interestingly, the interaction of TSH and BMI also appeared statistical significance in both male and female subjects: predicting total cholesterol and LDL cholesterol levels in males (both *P* < 0.05), while predicting TG levels in females (*P* < 0.01) (Table S1).

### 3.4. The Association between TH and Lipid Profile in the Normal-Weight and Overweight/Obese Groups

Since BMI modulates the interaction between thyroid parameters and lipid profile, we further checked whether the association between thyroid parameters and lipid profile varied in subjects with different BMI. We performed linear regression analyses separately in the normal-weight and overweight/obese groups (Table 3). In the normal-weight and overweight/obese groups, the subjects were further sequentially assigned to tertiles according to their plasma FT3 (Table 4), FT4 (Table 5), or TSH (Table 6) levels.

A positive correlation between FT3 and TG levels was only significant in the normal-weight subjects (*β* = 0.145 ± 0.071, *P* < 0.05; Table 3), with an increase in TG level across the tertiles **(***P* < 0.05; Table 4). In both normal-weight and overweight/obese groups, FT3 was positively associated with total cholesterol and LDL cholesterol levels (both *P* < 0.01, Table 3). In parallel, the higher FT3 tertiles (T2 and T3) had significantly elevated total cholesterol (*P* < 0.01) and LDL cholesterol levels (*P* < 0.01) compared to the lowest tertile (T1) in both normal-weight and overweight/obese groups (Table 4).

We then stratified the population according to their serum FT4 levels. A significant correlation between FT4 and TG (*β* = −0.071 ± 0.022, *P* < 0.01) as well as FT4 with HDL (*β* = 0.017 ± 0.006, *P* < 0.01) were only detected in the normal-weight subjects (both *P* < 0.01), but not in the overweight/obese group (Table 3). Similarly, significantly decreased plasma TG and increased HDL cholesterol levels (both *P* < 0.01) were observed in the highest tertile of FT4 only in the normal-weight group (Table 5).

A significant positive association between serum TSH levels and total cholesterol levels was only found in the normal-weight group (*β* = 0.110 ± 0.039, *P* < 0.01; Table 3). Indeed, significantly increased total cholesterol levels in the highest tertile of TSH were observed exclusively in the normal-weight group (*P* < 0.01; Table 6).

## 4. Discussion

Thyroid hormones influence key metabolic pathways which control energy balance by regulating energy storage and expenditure [5]. Overt thyroid dysfunctions are associated with alternations in lipid metabolism [2–5]. Recent studies have shown that changes in thyroid hormone levels, even within the physiological ranges, may contribute to the deteriorating of atherogenic lipid profile [6, 7, 9, 11, 12]. The present study with 1372 euthyroid adults demonstrated that FT3 was significantly and positively correlated with total and LDL cholesterol concentrations, whereas FT4 levels were negatively correlated with TG but positively associated with HDL cholesterol levels. Moreover, TSH levels were significantly and positively related to both TG and total cholesterol levels. Our findings were in line with several previous studies [6, 7, 12–16, 22, 23], showing that low normal FT4 [7, 9, 11–14] and high normal FT3 [6, 10] and TSH [11, 13] levels are associated with unfavorable lipid profile in euthyroid subjects. However, in obese euthyroid population, previous studies showed that some of these associations could not be identified [8] or disappeared after controlling for individuals' confounders including BMI [16]. Interestingly, when we performed linear regression analyses separately in the normal-weight and overweight/obese groups, we clearly reported that the associations between thyroid parameters and lipid parameters were not identical: the correlation between FT3 (or FT4) and TG and the correlation between TSH and total cholesterol were only significant in the normal-weight group, but not in individuals with BMI ≥ 25 kg/m^2^. The observations strongly indicate that body weight might interfere with the effect of TH on lipid profile.

The thyroid and adipose tissue are organs producing thyroid hormones and adipokines, respectively [24]. Both of them play central roles in the metabolism of the body. Several studies have shown that variations in TSH and thyroid hormones within normal ranges can influence body weight and subsequently alter lipid profile [25]. On the other hand, disturbed TH levels have been reported in obese subjects [25] and were also observed in subjects with BMI ≥ 25 kg/m^2^ in the current study. However, the influence of body weight on the interaction of TH and lipid profile has not been clearly clarified in the literature. Importantly, we showed that serum FT4 levels and BMI had interactions in predicting serum TG levels, whereas FT3 and BMI had interactions in predicting HDL cholesterol levels in euthyroid population. Such interactions remained significant and even more pronounced when we performed gender-based interaction analyses: TSH and BMI reached significance in predicting total cholesterol and LDL cholesterol levels in males, as well as TG levels in females.

As generally accepted, obesity is closely associated with dyslipidemia, manifested as elevated fasting and postprandial TG in combination with the preponderance of small dense LDL and low HDL cholesterol [26]. Obesity increases free fatty acid (FFA) fluxes to the liver, adipose tissue, and skeletal muscle, which leads to alter expression of lipoprotein lipase (LPL) activity and hampers lipolysis and TG accumulation and transport, subsequently causes dyslipidemia [26]. It is currently unknown how BMI interferes with the effect of thyroid hormones on lipid profile, and it is possible that certain adipokines, such as leptin, play a role via hypothalamic-pituitary-thyroid axis [27, 28]. Although changes in TH levels within normal ranges have independent influence on lipid profile in normal-weight subjects, such impact might be neglectable in overweight/obese individuals. Thus, the current observations might be clinical relevant regarding strategies for individualized lipid control. In normal-weight subjects, TH levels within normal ranges should be taken into account: low normal FT3, TSH levels, and high normal FT4 levels might be beneficial for lipid control. However, in overweight/obese subjects, weight loss absolutely takes priority. The disturbed levels of TH in obesity can be reversed after weight loss [29] and might have additional impact on lipid management.

The novelty of the study is that, to the best of our knowledge, we are the first to identify the role of BMI in modulating the effect of thyroid hormones on lipid profile in euthyroid adults. However, our study has several limitations. First, due to the small sample size of obese population, we could not compare overweight and obese subjects separately in our analyses. Second, lacking of smoking status information impeded us to identify the effect of smoking habit on such relation. Third, it would be ideal to initially exclude the subjects on lipid-lowering therapy, although we estimate that this percentage is very low in our study population. Fourth, as a cross-sectional study, the causal relationship could not be inferred. Further studies are needed to clarify whether adjusting certain TH parameters in euthyroid normal-weight subjects do bring benefits in maintaining lipid homeostasis.

In conclusion, in euthyroid adults, low normal FT4 levels, high normal FT3, and TSH levels are associated with less favorable lipid parameters. BMI modulates the effect of thyroid hormones on lipid metabolism. The findings that the associations between TH and lipid parameters are not identical in normal-weight and overweight/obese subjects provide clues for individualized lipid-lowering therapy in clinic.

## Supplementary Material

Table S1: The interaction between BMI and thyroid parameters in modulating lipids profile.

## Figures and Tables

**Table 1:** General, anthropometric, hormonal, and metabolic parameters of the study population.

	18.5 ≤ BMI< 25	BMI ≥ 25	*P* value
	(*n* = 820)	(*n* = 552)	
General characteristics			
Age (years)	48.33 ± 9.28	48.64 ± 8.34	0.525
Height (cm)	167.56 ± 7.05	170.61 ± 6.08	<**0.001**^∗∗∗^
Body weight (kg)	63.20 ± 8.00	79.52 ± 8.28	<**0.001**^∗∗∗^
BMI (kg/m^2^)	22.43 ± 1.70	27.28 ± 1.96	<**0.001**^∗∗∗^
Lipid profile			
TG (mmol/L)	1.27(0.87–1.89)	1.90 (1.32–2.86)	<**0.001**^∗∗∗^
Total cholesterol (mmol/L)	4.82 ± 0.96	4.95 ± 0.94	<**0.05**^∗^
HDL cholesterol (mg/L)	13.21 ± 3.34	11.64 ± 2.95	<**0.001**^∗∗∗^
LDL cholesterol (mg/L)	29.53 ± 8.18	29.93 ± 8.18	0.386
TSH and thyroid hormones			
FT3 (pmol/L)	4.11 ± 0.57	4.29 ± 0.58	<**0.001**^∗∗∗^
FT4 (pmol/L)	12.62 ± 1.70	12.41 ± 1.54	<**0.05**^∗^
TSH (mIU/L)	1.66 ± 0.86	1.58 ± 0.85	0.074

Subjects on thyroid medications, with TPO antibodies, anti-thyroglobulin antibodies above clinical cutoff, and with TSH levels outside the reference range, were excluded from further analyses. We stratified the study group according to BMI levels: 18.5 ≤ BMI< 25 kg/m^2^: normal-weight group; BMI ≥ 25 kg/m^2^: overweight/obese group. All continuous parameters were presented as mean ± standard deviation. For skewness distribution data, median and interquartile range was used. *P* values were calculated from one-way analysis of variance. BMI: body mass index; FT3: free triiodothyronine; FT4: free thyroxine; TSH: thyrotropin; TG: triglycerides; HDL: high-density lipoprotein; LDL: low-density lipoprotein. Significant associations are indicated in bold. *P* < 0.05 was considered statistically significant. ^∗^*P* < 0.05, ^∗∗∗^*P* < 0.001.

**Table 2:** The association between lipid profile and thyroid parameters.

Dependent variable	FT3		FT4		TSH	
	*β* ± SE	*P*	*β* ± SE	*P*	*β* ± SE	*P*
TG	0.075 ± 0.087	0.392	−0.087 ± 0.029	**0.003** ^∗∗^	0.145 ± 0.056	**0.010** ^∗^
CHOL	0.176 ± 0.046	**0.0002** ^∗∗∗^	0.005 ± 0.016	0.761	0.094 ± 0.030	**0.002** ^∗∗^
HDL-C	0.029 ± 0.015	0.051	0.013 ± 0.005	**0.007** ^∗∗^	−0.002 ± 0.010	0.825
LDL-C	0.161 ± 0.040	*P* < 0.0001^∗∗∗^	0.016 ± 0.013	0.237	0.039 ± 0.026	0.137

Reported values are betas ± standard error and results from linear regression analysis with lipid parameters as dependent variables and thyroid parameters as independent variables. Betas are scaled and adjusted for age, gender, and BMI. CHOL: total cholesterol; HDL-C: high-density lipoprotein cholesterol; LDL-C: low-density lipoprotein cholesterol. Significant associations are indicated in bold. *P* < 0.05 was considered statistically significant. ^∗^*P* < 0.05, ^∗∗^*P* < 0.01, and ^∗∗∗^*P* < 0.001.

**Table 3:** The association between lipids profile and thyroid parameters in different BMI subgroups.

FT3						FT4				TSH					
	BMI < 25		BMI ≥ 25		*P* interaction	BMI < 2		BMI ≥ 25		*P* interaction	BMI< 25		BMI ≥ 25		*P* interaction
	*β* ± SE	*P*	*β* ± SE	*P*		*β* ± SE	*P*	*β* ± SE	*P*		*β* ± SE	*P*	*β* ± SE	*P*	
TG	0.145 ± 0.071	**0.041** ^∗^	0.076 ± 0.187	0.687	0.687	−0.071 ± 0.022	**0.002** ^∗∗^	−0.113 ± 0.069	0.101	**0.023** ^∗^	0.078 ± 0.045	0.085	0.245 ± 0.125	0.051	0.138
CHOL	0.172 ± 0.061	**0.005** ^∗∗^	0.205 ± 0.070	**0.004** ^∗∗^	0.171	0.004 ± 0.020	0.822	0.005 ± 0.026	0.840	0.769	0.110 ± 0.039	**0.005** ^∗∗^	0.068 ± 0.047	0.149	0.193
HDL-C	0.010 ± 0.020	0.613	0.041 ± 0.022	0.058	0.040^∗^	0.017 ± 0.006	**0.010** ^∗∗^	0.010 ± 0.008	0.220	0.258	0.008 ± 0.013	0.548	−0.018 ± 0.014	0.203	0.105
LDL-C	0.158 ± 0.053	**0.003** ^∗∗^	0.177 ± 0.061	**0.004** ^∗∗^	0.054	0.007 ± 0.017	0.695	0.031 ± 0.023	0.177	0.283	0.063 ± 0.033	0.061	0.003 ± 0.041	0.944	0.103

We stratified the study group according to BMI levels: BMI < 25 kg/m^2^: normal-weight group; BMI ≥ 25 kg/m^2^: overweight/obese group. Reported values are betas ± standard error and results from linear regression analysis with lipid parameters as dependent variables and thyroid parameters as independent variables. Betas are scaled and adjusted for age and sex. *P* for interaction results from linear regression analysis with FT3^∗^BMI (or FT4^∗^BMI, TSH^∗^BMI) as independent variable and lipid profile as dependent variables. Significant associations are indicated in bold. *P* < 0.05 was considered statistically significant. ^∗^*P* < 0.05, ^∗∗^*P* < 0.01.

**Table 4:** Lipid profile in different FT3 and BMI categories.

	BMI < 25				BMI ≥ 25			
	Group 1 FT3 T1	Group 2 FT3 T2	Group 3 FT3 T3	*P*	Group 4 FT3 T1	Group 5 FT3 T2	Group 6 FT3 T3	*P*
TG	1.05 (0.74–1.56)	1.29 (0.90–1.84)	1.55 (1.02–2.17)	**0.041** ^∗^	1.75 (1.13–2.60)	1.88 (1.34–2.90)	2.09 (1.40–3.06)	0.687
CHOL	4.72 ± 0.96	4.81 ± 0.95	4.92 ± 0.95	**0.005** ^∗∗^	4.82 ± 0.87	4.97 ± 0.98	5.06 ± 0.94	**0.004** ^∗∗^
HDL-C	1.36 ± 0.33	1.32 ± 0.34	1.28 ± 0.32	0.613	1.16 ± 0.31	1.14 ± 0.28	1.19 ± 0.30	0.058
LDL-C	2.86 ± 0.81	2.93 ± 0.81	3.06 ± 0.83	**0.003** ^∗∗^	2.85 ± 0.77	3.03 ± 0.86	3.09 ± 0.82	**0.004** ^∗∗^

We stratified the study group according to BMI levels: BMI < 25 kg/m^2^: normal-weight group; BMI ≥ 25 kg/m^2^: overweight/obese group. Then, we stratified each group according to serum FT3 levels; and all continuous parameters were presented as mean ± standard deviation, after adjustment for age and gender. For skewness distribution data, median and interquartile range was used. *P* values were calculated from ANOVA (analysis of variance, between categories), after adjustment for age and gender. Significant associations are indicated in bold. *P* < 0.05 was considered statistically significant. ^∗^*P* < 0.05, ^∗∗^*P* < 0.01.

**Table 5:** Lipid profile in different FT4 and BMI categories.

	BMI < 25				BMI ≥ 25			
	Group 1 FT4 T1	Group 2 FT4 T2	Group 3 FT4 T3	*P*	Group 4 FT4 T1	Group 5 FT4 T2	Group 6 FT4 T3	*P*
TG	1.30 (0.93–2.02)	1.36 (0.92–1.90)	1.16 (0.82–1.71)	**0.002** ^∗∗^	2.04 (1.36–3.11)	2.18 (1.41–3.29)	1.68 (1.20–2.35)	0.101
CHOL	4.80 ± 0.95	4.81 ± 0.92	4.84 ± 1.00	0.822	4.92 ± 0.96	5.01 ± 0.88	4.92 ± 0.96	0.840
HDL-C	1.30 ± 0.33	1.31 ± 0.32	1.35 ± 0.34	**0.010** ^∗∗^	1.14 ± 0.26	1.14 ± 0.30	1.22 ± 0.32	0.220
LDL-C	2.94 ± 0.80	2.95 ± 0.78	2.98 ± 0.88	0.695	2.96 ± 0.85	2.97 ± 0.78	3.04 ± 0.82	0.177

We stratified the study group according to BMI levels: BMI < 25 kg/m^2^: normal-weight group; BMI ≥ 25 kg/m^2^: overweight/obese group. Then, we stratified each group according to serum FT4 levels; all continuous parameters were presented as mean ± standard deviation, after adjustment for age and gender. For skewness distribution data, median and interquartile range was used. *P* values were calculated from ANOVA (analysis of variance, between categories), after adjustment for age and gender. Significant associations are indicated in bold. *P* < 0.05 was considered statistically significant. ^∗∗^*P* < 0.01.

**Table 6:** Lipid profile in different TSH and BMI categories.

	BMI < 25				BMI ≥ 25			
	Group 1 TSH T1	Group 2 TSH T2	Group 3 TSH T3	*P*	Group 4 TSH T1	Group 5 TSH T2	Group 6 TSH T3	*P*
TG	1.21 (0.82–1.88)	1.32 (0.93–1.95)	1.23 (0.88–1.84)	0.085	1.30 (1.81–2.76)	1.86 (1.27–2.66)	2.01 (1.37–3.18)	0.051
CHOL	4.71 ± 0.90	4.81 ± 0.95	4.93 ± 1.01	**0.005** ^∗∗^	4.87 ± 0.96	4.91 ± 0.89	5.08 ± 0.94	0.149
HDL-C	1.31 ± 0.32	1.31 ± 0.34	1.35 ± 0.34	0.548	1.15 ± 0.30	1.19 ± 0.29	1.15 ± 0.30	0.203
LDL-C	2.88 ± 0.76	2.96 ± 0.82	3.03 ± 0.87	0.061	2.96 ± 0.83	3.01 ± 0.77	3.00 ± 0.86	0.944

We stratified the study group according to BMI levels: BMI < 25 kg/m^2^; normal-weight group; BMI ≥ 25 kg/m^2^: overweight/obese group. Then, we stratified each group according to serum TSH levels; all continuous parameters were presented as mean ± standard deviation, after adjustment for age and gender. For skewness distribution data, median and interquartile range was used. *P* values were calculated from ANOVA (analysis of variance, between categories), after adjustment for age and gender. Significant associations are indicated in bold. *P* < 0.05 was considered statistically significant. ^∗∗^*P* < 0.01.
