# Supplementary material for: BMI Modulates the Effect of Thyroid Hormone on Lipid Profile in Euthyroid Adults
Source: Int J Endocrinol. 2017 Aug 13;2017:8591986. doi: 10.1155/2017/8591986 (PMC5572619; doi:10.1155/2017/8591986)
Supplement: Supplementary file 1 — Table S1: The interaction between BMI and thyroid parameters in modulating lipids profile. [file 8591986.f1.pdf]

Table S1 The interaction between BMI and thyroid parameters in modulating lipids profile

$P_{interaction}$	FT3*BMI		FT4*BMI		TSH*BMI	
	Male	Female	Male	Female	Male	Female
TG	0.605	0.539	0.239	**0.039***	0.429	**0.001****
CHOL	0.756	0.466	0.411	0.547	**0.036***	0.987
HDL-C	**0.023***	0.203	0.538	0.127	0.583	0.083
LDL-C	0.420	0.438	0.119	0.689	**0.019***	0.844

P for interaction results from linear regression analysis with FT3*BMI（or FT4*BMI, TSH*BMI）as independent variable and lipids profile as dependent variables, after multiple adjustment for age, BMI, FT3 (or FT4, TSH). Significant associations are indicated in bold.
p<0.05 was considered statistically significant.
*p<0.05, ** p<0.01, ***p<0.001
